# Functional Polymorphisms of Interferon-gamma Affect Pneumonia-Induced Sepsis

**DOI:** 10.1371/journal.pone.0087049

**Published:** 2014-01-24

**Authors:** Ding Wang, Xuan Zhong, Dongjian Huang, Rui Chen, Guibin Bai, Qing Li, Bolan Yu, Yong Fan, Xiaofang Sun

**Affiliations:** 1 Key Laboratory for Major Obstetric Diseases of Guangdong Province, Key Laboratory of Reproduction and Genetics of Guangdong Higher Education Institutes, Experimental Department of Institute of Gynecology and Obstetrics, The Third Affiliated Hospital of Guangzhou Medical University, Guangzhou, China; 2 The department of intensive care unit, The Third Affiliated Hospital of Guangzhou Medical University, Guangzhou, China; 3 Reproductive Department, The Third Affiliated Hospital of Guangzhou Medical University, Guangzhou, China; University of Cincinnati, United States of America

## Abstract

**Objective:**

Sepsis is an inflammatory syndrome caused by infection, and both its incidence and mortality are high. Because interferon-gamma (IFN-γ) plays an important role in inflammation, this work assessed IFN-γ single nucleotide polymorphism (SNPs) that may be associated with sepsis.

**Methods:**

A total of 196 patients with pneumonia-induced sepsis and 213 age- and sex-matched healthy volunteers participated in our study from July 2012 to July 2013 in Guangzhou, China. Patient clinical information was collected. Clinical pathology was assessed in subgroups defined based on clinical criteria, APACHE II (acute physiology and chronic health evaluation) and SOFA (sepsis-related organ failure assessment) scores and discharge rate. Four functional SNPs, −1616T/C (rs2069705), −764G/C (rs2069707), +874A/T (rs2430561) and +3234C/T (rs2069718), were genotyped by Snapshot in both sepsis patients and healthy controls. Pearson’s chi-square test or Fisher’s exact test were used to analyze the distribution of the SNPs, and the probability values (P values), odds ratios (OR) and 95% confidence intervals (CIs) were calculated.

**Results:**

No mutations in the IFN-γ −764G/C SNP were detected among the participants in our study. The +874A/T and +3234C/T SNPs were in strong linkage disequilibrium (LD) (r^2^ = 0.894). The −1616 TC+TT, +874 AT+AA genotype and the TAC haplotype were significantly associated with sepsis susceptibility, while the CTT haplotype was associated with protection against sepsis incidence. Genotype of −1616 TT wasn’t only protective against severity of sepsis, but also against higher APACHE II and SOFA scores as +874 AA and +3234 CC. The TAC haplotype was was protective against progression to severe sepsis either.

**Conclusion:**

Our results suggest that functional IFN-γ SNPs and their haplotypes are associated with pneumonia-induced sepsis.

## Introduction

Sepsis can be defined as a systemic inflammatory response syndrome caused by infectious pathogens [1], while severe sepsis is sepsis combined with acute organ dysfunction, including hypoperfusion and hypotension [2]. The mortality rate from sepsis is 10–20%, and the mortality rate from severe sepsis is 20–50% [1]; these rates correspond to those reported in surveys of Chinese epidemics [3]. Because the disease is severely life threatening, the pathogenesis, prognostic factors and modes of therapy are of great interest to both clinicians and academic researchers. Recently, some studies showed that the individual genetic background is associated with the personal inflammatory response and also influences the pathology. Genes with related polymorphisms include the antigen recognition pathway protein [4], [5], proteins associated with blood biochemistry [6], pro- [7], [8] and anti-inflammatory cytokines [9], [10] and others.

IFN-γ, a member of the interferon family, plays an important role in inflammation and acquired immunity in infection [11]. Initially, IFN-γ was presumed to only interfere with viral replication [12], but subsequent experimental studies showed that it also protects the individual against bacterial [13], [14] and fungal pathogens [15]. The most important function of IFN-γ is its macrophage-activating function, which up-regulates the expression of the major histocompatibility complexes (MHC) I [16], [17] and II [18], [19], which are involved in the antigen processing and presentation pathways. IFN-γ also mediates functions such as leukocyte attraction [16], [20], maturation and differentiation, natural killer (NK) cell activity [21] and immunoglobulin (Ig) production and class switching in B cells [16]. In sepsis, IFN-γ is an important regulator of infection and inflammation; it is released in small amounts during the initial period of infection, and the amount released increases during the inflammation period [22]–[24]. It is postulated that suppressing IFN-γ to near normal levels during the early period of inflammation may benefit the host by preventing bacteria outflow [22]. Increased IFN-γ levels during the later period [22]–[24] may be beneficial by stimulating the body’s defense system [16].

The IFN-γ gene is located on chromosome 12 q14, and several SNPs in this gene have reportedly been associated with immunologic diseases such as aplastic anemia [25], hepatitis infection [26]–[29], systemic lupus erythematosus [30], [31], and asthma [32], [33]. Some of the disease-associated SNPs are functional. The SNPs in the 5′ untranslated regions (UTR) are translation-level regulators [26], [31], [34], [35], and some SNPs in the introns may function to modify mRNA expression [36]–[39]. Both the minor allele frequency (MAF) and the influence of the SNPs on disease pathology vary among populations. In addition to IFN-γ participating in the key immune response and sepsis development, its SNPs are functional and are related to immunologic disease. There have been few reports in the literature regarding the epidemiology of IFN-γ SNPs in relation to sepsis, with the exception of one study examining the association between intron 1 polymorphisms and trauma due to sepsis [40].

We focused on interpreting the relationship between pneumonia-induced sepsis and four functional SNPs of IFN-γ; these included two SNPs in the 5′ UTR −1616T/C (rs2069705) and −764G/C (rs2069707), as well as two SNPs in the introns +874A/T (rs2430561, intron1) and +3234C/T (rs2069718, intron 3). To the best of our knowledge, this study is the first to assess the potential implication of IFN-γ functional genotypes and haplotypes on the incidence, development and outcome of pneumonia-induced sepsis.

## Materials and Methods

### Study Population

In this study, 196 patients with pneumonia-induced sepsis were enrolled within 24 hours of admission to the intensive care unit (ICU) at the Third Affiliated Hospital of Guangzhou Medical University (Guangzhou, China) from July 2012 to July 2013. The patients in the experimental group have signed their names by Chinese on written informed consent, and agreed that their peripheral blood will be used for genotyping and collection of other clinical information. The peripheral blood sample of each individual was collected by routine examination, and the examination result was entered into our study database supplemented with the information of sex, age and the clinical description collected from the physician. For the control group, 213 health examination volunteers without infection and sepsis, with sex and age matched, were selected from our hospital database, and the blood samples were obtained from the clinical laboratory. The volunteers have agreed on a verbal consent that their peripheral blood will be used for harmless, non-profit purpose only and their personal information will be kept confidential upon the health examination; therefore, we didn’t re-connect them for the written consent. This practice of verbally informed consent was reviewed and approved by the Ethics Committee. The control group entries in our database included no personal information but sex, age and peripheral blood sample number only. The present study was approved by the Ethics Committee of The Third Affiliated Hospital of Guangzhou Medical University. The exclusion criteria were <18 years of age, primary site of infection other than the lungs, un-drainable surgical source of sepsis, missing informed consent forms and patients with immunosuppression of any etiology, including cancer, current immunosuppressive therapy or chemotherapy, human immunodeficiency virus (HIV) infection, liver insufficiency and severe chronic renal disease with dialysis therapy. The severe group included those who experienced organ dysfunction, and the severity of organ dysfunction was graded according to the APACHE II and SOFA indexes.

### SNP Genotype

The four IFN-γ SNPs(764G/C, −1616T/C, +874A/T and +3234C/T), as the gene expression involved based on the other functional experiments, were selected. Genomic DNA was extracted from peripheral blood using QIAamp DNA Blood Mini Kit (Qiagen, Dusseldorf, Germany) according to the manual and stored at −20°C before use. The primers (Table 1) for polymerase chain reaction (PCR) amplification and Snapshot extension reactions were designed by the Primer Premier 5 program and the sequence from the gene bank of the National Center for Biotechnology Information (NCBI). Every PCR amplicon was confirmed by agarose electrophoresis for fragment size and detailed by sequencing on an ABI 3130XL sequencer (Applied Biosystems, ABI, California, USA). PCR was performed using the Hot Star Taq kit (Qiagen). PCR products were purified using shrimp alkaline enzyme (SAP) (Promega, Wisconsin, USA) and exonuclease I (EXO I) (EpiCentre, Palmerston North, New Zealand) according to the manufacturers’ instructions and were used as a template for extension. Extension was performed using a commercial kit for Snapshot Multiplex reaction (ABI), and the products were purified using SAP (Promega) and loaded onto an ABI 3130XL sequencer for sequencing. The raw data from the ABI 3130XL sequencer were subjected to analysis with GeneMapper 4.0 (ABI).

**Table 1:** Primers for PCR and Snapshot.

	Primer 5′–3′	Size (bp)
PCR		
−1616T/C	F:CCTAGCACTTTATGAGGATTACC	
	R:GTTATGGGGCAAACTTGATTC	176
−764G/C	F:GTCTCAAACTCCTGACCTTGT	
	R:CTTCAGTATGCATCAATATACTACAT	187
+874A/T	F:TACATCTACTGTGCCTTCCTG	
	R:CATTATTTGTTTAAAACTTAGCTGT	195
+3234C/T	F:TGGTGAGTAGCCATAGTGTTCC	
	R:ACTTTTCCAGTACCCTGCCTT	171
Snapshot		
−1616C/T	ATCTAGCTATATGATTGTGAGTTA	24
−764G/C	TGGAACTCCCCCTGGGAATATTCT	24
+874A/T	TTATTCTTACAACACAAAATCAAATC	26
+3234C/T	GAGGAAGGTAAATGGTCCACAT	22

### Statistical Analysis

The demographic variables and differences between the sepsis patients and controls were tested using Pearson’s chi-square test (for categorical variables) or Student’s t-test (for continuous variables). As there were no mutations for IFN-γ −764G/C, Hardy–Weinberg equilibrium (HWE) for allele, genotype and haplotype of the other three IFN-γ SNPs (−1616T/C, +874A/T and +3234C/T) were compared between cases and controls and between groups of cases using the Pearson chi-square test or Fisher’s exact test. For these tests, P values of <0.05 were considered to be statistically significant. The Bonferroni correction was applied for SNP allele and genotype analysis; when the three SNPs were regarded as two independent factors, the significance threshold was adjusted to <0.025 and >0.025, with <0.05 considered as the margin. For every comparison, odds ratios (OR) with respective confidence intervals (95% CI) were calculated, and for significant and marginal P values, OR >1 and 95% CI >1 indicated susceptibility, while OR <1 and 95% CI <1 indicated protection. However, the OR and 95% CI were not available for all acceptable P values because they require at least one case, as otherwise the value of the weight variable is zero. Multivariate logistic regression models were used to assess the incidence, development and outcome of sepsis, controlling for age and sex as potential risk factors. All risk factors were defined categorically, and all tests were adjusted for age and sex. SPSS 19.0 (SPSS Inc., Chicago, IL, USA) was used for statistical calculations, and the online software SHESIS was used to calculate linkage disequilibrium (LD).

## Results

### Characteristics and Grouping of the Study Population

The patients in our study consisted of 196 individuals from Southern China who had at least two conditions meeting the criteria of systemic inflammatory response syndrome (SIRS), including 107 individuals with acute organ dysfunction (including hypoperfusion and hypotension) or shock who were categorized as having severe sepsis. All patients were treated for sepsis in the ICU and received specific antimicrobial therapy and supportive care. The patients’ clinical information and characteristics of the groups are shown in Table 2. The sex ratio was nearly 1∶1, the mean age was 64.09 years and the standard deviation was 20.67 (Table 2). The control group included 213 healthy age- and sex- matched volunteers, and the t test showed no significant differences between the sepsis group and the control group in terms of age (P = 0.905) or sex (P = 0.169) (Table 2). For the groups with sepsis and severe sepsis, there were significant differences in age and APACHE II and SOFA scores according to the t test (P<0.0001) (Table 2). The APACHE II and SOFA scores were graded according to clinical pathology, and the APACHE II 20 and SOFA 10 scores were used to divide patients with sepsis into the sepsis and severe sepsis groups, with the two groups exhibiting significantly different scores according to the t test. The group with the higher score was older, but there was no difference in terms of sex distribution (Table 2). Among all patients with sepsis, 109 recovered and were discharged, while 87 died. The mortality rate among patients with sepsis was 40%, and the mortality rate was 44.86% among patients with severe sepsis. Death was significantly associated with the APACHE II score (P = 0.012) and marginally associated with the SOFA score (P = 0.06), but it was not associated with sex or age (Table 2).

**Table 2:** Characteristics and grouping of the study population.

	Sepsis(n = 196)	Control(n = 213)	P value	Sepsis(n = 89)	serve sepsis(n = 107)	P value	SOFA< = 10(n = 104)	SOFA>10(n = 92)	P value	APACHE< = 20(n = 94)	APACHE>20(n = 102)	P value	Discharged(n = 109)	Death(n = 87)	P value
Age (mean±SD)^a^	64.09±20.67	63.57±20.68	0.905	57.02±21.43	70.04±18.06	<0.0001	61.04±21.18	67.58±19.61	0.026	56.87±22.19	70.55±16.86	0.001	62.46±20.62	66.49±20.85	0.182
deathe case(Mortality%)^b^	87 (44.39)			36 (40.45)	51 (47.66)	0.01	36 (40.45)	51 (47.66)	0.01	36 (38.30)	51 (50.00)	0.006			
APACHE II (mean±SD)^a^	20.43±5.13			17.93±7.28	22.52±7.69	<0.0001							19.25±7.79	22.09±7.71	0.012
SOFAmax (mean±SD)^a^	9.88±7.83			8.40±4.78	11.12±5.10	<0.0001							9.27±5.19	10.67±5.06	0.06
Female (%)	86 (43.88)	110 (51.64)		44 (49.44)	42 (39.25)		45 (43.27)	41 (44.57)		45 (47.87)	41 (40.20)		49 (44.95)	37 (42.53)	
Male(%)^b^	110 (56.12)	103 (48.36)	0.169	45 (50.56)	65 (60.75)	0.15	59 (56.73)	51 (55.43)	0.943	49 (52.13)	61 (59.80)	0.42	60 (55.05)	50 (57.47)	0.73
Pathgeno															
G+ bacteria (%)	111 (56.63)														
G− bacteria (%)	120 (61.22)														
Fungi (%)	42 (21.43)														

a means statistics by Student’s t-test.

b means statistics by Pearson’s chi-square test.

### Association of the IFN-γ SNPs with Risk of Sepsis

While the +784 and +3234 SNPs exhibited strong equilibrium, their linkages with −1616 were not obvious (Table 3). For monofactorial analysis, the threshold of the P value was 0.05, though for the Bonferroni correction, considering that two of the three SNPs showed strong LD, the threshold of the P value was adjusted to 0.025, and we accepted P values <0.05 and >0.025 as being marginally significant. Although the age and sex distribution did not differ between patients with sepsis and healthy controls, we adjusted the genotype results by age and sex in order to increase confidence in our results. The genotype frequency of the three positions indicated different trends for sepsis risk (Table 3). The −1616 SNP was significantly associated with sepsis risk, and the frequency of the TC (P = 0.0045) [OR and 95% CI: 1.99 (1.31–3.01)] and TC+TT (P = 0.0024) [1.84 (1.24–2.73)] genotype were greater among patients with sepsis compared with healthy controls (Table 3). The genotype frequency of +874 indicated a trend (P  = 0.013) of higher frequency of TA+AA in the sepsis group [1.68 (1.11–2.54)]; the A allele was significantly associated with sepsis susceptibility (P = 0.024) [1.49 (1.05–2.12)] (Table 3). There were no significant associations in the +3234 analysis for either genotype (P = 0.18 and 0.067) or allele (P = 0.2) (Table 3). Following the law of linkage disequilibrium, the −1616, +874 and +3234 loci formed three dominant haplotypes, which were CTT, TAC and TTT. CTT conferred protection against sepsis (P = 0.009) [0.66 (0.49–0.90)], but TAC was associated with susceptibility to sepsis (P = 0.022) [1.51 (1.06–2.16)] (Table 3).

**Table 3:** Association of the IFN-γ SNPs with risk of sepsis.

	Sepsis (%)	Control (%)	OR(95%CI)	P value	r^2^	
					−1616T/C	+874 A/T
−1616 T/C						
CC	83 (42.3)	121 (56.8)	1.00 (reference)			
TC	98 (50.0)	74 (34.7)	**1.99 (1.31–3.01)**			
TT	15 (7.7)	18 (8.5)	1.22 (0.58–2.57)	**0.0045**		
TC+TT	113 (57.7)	92 (43.2)	**1.84 (1.24–2.73)**	**0.0024**		
C	264 (67.3)	316 (74.2)	1.00 (reference)			
T	128 (32.7)	110 (25.8)	0.72 (0.53–0.97)	0.032		
+874 A/T					0.52	
TT	116 (59.2)	150 (70.4)	1.00 (reference)			
TA	71 (36.2)	56 (26.3)	1.68 (1.09–2.58)			
AA	9 (4.6)	7 (3.3)	1.70 (0.61–4.78)	0.046		
TA+AA	80 (40.8)	63 (29.6)	**1.68 (1.11–2.54)**	**0.013**		
T	303 (77.3)	356 (83.6)	1.00 (reference)			
A	89 (22.7)	70 (16.4)	**1.49 (1.05–2.12)**	**0.024**		
+3234 C/T					0.595	0.894
TT	117 (69.7)	145 (68.1)	1.00 (reference)			
TC	71 (36.2)	60 (28.2)	1.49 (0.97–2.27)			
CC	8 (4.1)	8 (3.8)	1.26 (0.45–3.50)	0.18		
TC+CC	79 (40.3)	68 (32.0)	1.46 (0.97–2.20)	0.067		
T	305 (77.8)	350 (82.2)	1.00 (reference)			
C	87 (22.2)	76 (17.8)	1.31 (0.93–1.85)	0.2		
Haplotype						
C T T	262 (66.8)	311.85 (73.2)	**0.66 (0.49–0.90)**	**0.009**		
T A C	87 (22.2)	65.85 (15.5)	**1.51 (1.06–2.16)**	**0.022**		
T T T	41 (10.5)	35.09 (8.2)	1.27 (0.79–2.03)	0.33		

Values in bold and underline indicate statistical significance.

### Combination Analysis of the IFN-γ SNPs and Sepsis Development by Clinical Grouping

We created four divisions within the sepsis patients in order to interpret the relationship between sepsis development and IFN-γ SNPs (Table 4). The group of patients with severe sepsis according to the APACHE II and SOFA scores was divided according to sepsis progression, and patients were also divided based on final outcome, i.e., survival. There weren’t any sense result in the dominant mutations analysis. The +874T allele was significant protective against progression of severe sepsis (P = 0.02) [0.57 (0.35–0.91)], while +3234C were marginally (P = 0.04) [0.60 (0.37–0.98)] (Table 4). The −1616 TT genotype was also protective against sepsis progression (P<0.001) [0.06 (0.01–0.50)] and against higher APACHE II (P<0.0001) and SOFA (P = 0.001) [0.09 (0.01–0.75)] scores (Table 4). The +874 AA and +3234 CC genotype didn’t appearance in the higher score group, and the trend was significant (Table 4). To our surprise, these results were opposite of the haplotype results regarding sepsis risk, as the CTT haplotype as associated with a marginally higher risk of sepsis progression (P = 0.054) [1.51 (0.99–2.31)], though the TAC haplotype was associated with a lower risk of progression to severe sepsis (P = 0.038) [0.60 (0.37–0.98)] (Table 4). The other tests for sepsis development were rejected, and there was no accepted test of genotype or haplotype for sepsis outcome (Table 4).

**Table 4:** Combination analysis of the IFN-γ SNPs and sepsis development by clinical grouping.

	Sepsis(%)	Severesepsis (%)	OR(95%CL)	Pvalue	APACHE < = 20 (%)	APACHE >20 (%)	OR(95%CL)	Pvalue	SOFA < = 10 (%)	SOFA >10 (%)	OR(95%CL)	Pvalue	Discharged (%)	Death(%)	OR(95%CL)	P value
−1616 T/C																
CC	36 (40.5)	47 (43.9)	1.00 (reference)		41 (44.1)	42 (41.2)	1.00 (reference)		45 (43.3)	38 (41.3)	1.00 (reference)		41 (37.6)	42 (48.3%)	1.00 (reference)	
TC	39 (43.8)	59 (55.1)	1.25 (0.67–2.34)		38 (40.9)	60 (58.8)	1.65 (0.88–3.10)		45 (43.3)	53 (57.6)	1.39 (0.77–2.54)		60 (55.1)	38 (43.7%)	0.68 (0.37–1.25)	
TT	14 (15.7)	1 (0.9)	**0.06 (0.01–0.50)**	**<0.001**	15 (16)	0	**NA**	**<0.0001**	14 (13.5)	1 (1.1)	**0.09 (0.01–0.75)**	**0.001**	8 (7.3)	7 (8.3)	0.98 (0.31–3.07)	0.44
TC+TT	53 (59.5)	60 (56.1)	0.94 (0.51–1.72)	0.84	53 (56.4)	60 (58.8)	1.18 (0.65–2.16)	0.58	59 (56.7)	54 (58.7)	1.10 (0.61–1.97)	0.75	68 (62.4)	42 (50)	0.72 (0.40–1.29)	0.27
C	111 (62.4)	153 (71.5)	1.00 (reference)		120 (63.8)	144 (70.6)	1.00 (reference)		135 (64.9)	129 (70.1)	1.00 (reference)		142 (65.1)	122 (70.1)	1.00 (reference)	
T	67 (37.6)	61 (28.5)	1.51 (0.99–2.31)	0.05	68 (36.2)	60 (29.4)	1.36 (0.89–2.07)		73 (35.1)	55 (29.9)	1.27 (0.83–1.94)	0.27	76 (34.9)	52 (29.9)	1.26 (0.82–1.93)	0.3
+874 A/T																
TT	47 (52.8)	69 (64.5)	1.00 (reference)		55 (58.5)	61 (59.8)	1.00 (reference)		62 (59.6)	54 (58.7)	1.00 (reference)		58 (53.2)	58 (66.7)	1.00 (reference)	
TA	34 (38.2)	37 (34.6)	0.77 (0.41–1.43)		30 (31.9)	41 (40.2)	1.28 (0.68–2.38)		33 (31.7)	38 (41.3)	1.30 (0.71–2.38)		45 (41.3)	26 (29.9)	0.63 (0.34–1.18)	
AA	8 (9)	1 (0.9)	0.19 (0.02–1.66)	0.18	9 (9.6)	0 (0)	**NA**	**0.012**	9 (8.7)	0 (0)	**NA**	**0.005**	6 (5.5)	3 (3.4)	1.45 (0.31–6.79)	0.35
TA+AA	42 (47.2)	38 (35.5)	0.70 (0.38–1.29)	0.25	39 (41.5)	41 (40.2)	1.08 (0.59–2.00)	0.8	42 (40.4)	38 (41.3)	1.09 (0.60–1.97)	0.77	51 (46.8)	29 (33.3)	0.65 (0.36–1.19)	0.16
A	50 (28.1)	39 (18.2)	1.00 (reference)		48 (25.5)	41 (20.1)	1.00 (reference)		51 (24.5)	38 (20.7)	1.00 (reference)		57 (26.1)	32 (18.4)	1.00 (reference)	
T	128 (71.9)	175 (81.8)	**0.57 (0.35–0.91)**	**0.02**	140 (74.5)	163 (79.9)	0.73 (0.46–1.19)	0.2	157 (75.5)	146 (79.3)	0.80 (0.50–1.29)	0.36	161 (73.9)	142 (81.6)	0.64 (0.39–1.04)	0.07
+3234 C/T																
TT	48 (53.9)	69 (64.5)	1.00 (reference)		56 (59.6)	61 (59.8)	1.00 (reference)		63 (60.6)	54 (58.7)	1.00 (reference)		59 (54.1)	58 (66.7)	1.00 (reference)	
TC	34 (38.2)	37 (34.6)	0.78 (0.42–1.45)		30 (31.9)	41 (40.2)	1.30 (0.70–2.42)		33 (31.7)	38 (41.3)	1.33 (0.73–2.42)		45 (41.3)	26 (29.9)	0.64 (0.35–1.20)	
CC	7 (7.9)	1 (0.9)	0.22 (0.02–2.01)	0.27	8 (8.5)	0 (0)	**NA**	**0.02**	8 (7.7)	0 (0)	**NA**	**0.009**	5 (4.6)	3 (3.6)	1.07 (0.22–5.15)	0.36
TC+CC	41 (46.1)	38 (35.5)	0.72 (0.39–1.33)	0.3	38 (40.4)	41 (40.2)	1.12 (0.61–2.06)	0.72	41 (39.4)	38 (41.3)	0.79 (0.41–1.51)	0.81	50 (45.9)	29 (33.3)	0.67 (0.37–1.23)	0.20
T	130 (73.0)	175 (81.8)	1.00 (reference)		142 (75.5)	163 (79.9)	1.00 (reference)		159 (76.4)	146 (79.3)	1.00 (reference)		163 (74.8)	142 (81.6)	1.00 (reference)	
C	48 (27.0)	39 (18.2)	0.60 (0.37–0.98)	0.04	46 (24.5)	41 (20.1)	0.78 (0.48–1.25)	0.3	49 (23.6)	38 (20.7)	0.84 (0.52–1.36)	0.49	55 (25.2)	32 (18.4)	0.67 (0.41–1.09)	0.11
Haplotype																
C T T	111 (61.7)	153 (71.5)	1.51 (0.99–2.31)	0.054	120 (63.2)	144 (70.6)	1.36 (0.89–2.08)	0.154	135 (64.3)	129 (70.1)	1.27 (0.83–1.94)	0.273	139 (63.8)	125 (71.8)	1.32 (0.86–2.04)	0.202
T A C	48 (26.7)	39 (18.2)	**0.60 (0.37–0.98)**	**0.038**	46 (24.2)	41 (20.1)	0.78 (0.48–1.25)	0.298	49 (23.3)	38 (20.7)	0.85 (0.52–1.36)	0.489	56 (25.7)	31 (17.8)	0.68 (0.41–1.11)	0.122
T T T	19 (10.6)	22 (10.3)	0.96 (0.50–1.84)	0.899	22 (11.6)	19 (9.3)	0.78 (0.41–1.48)	0.44	24 (11.4)	17 (9.2)	0.78 (0.41–1.50)	0.458	23 (10.5)	18 (10.4)	1.05 (0.55–2.01)	0.886

Values in bold and underline indicate statistical significance.

## Discussion

Sepsis, especially severe sepsis, is a great hazard to human health, as indicated by its increasing incidence and the higher than average mortality rates in intensive care units (ICU) [1], [41]. According to the American College of Chest Physicians (ACCP) and the Society of Critical Care Medicine (SCCM), sepsis is defined as having at least two criteria of systemic inflammatory response syndrome (SIRS) in addition to confirmed or suspected pathogenic infection. SIRS refers to a series of clinical symptoms involved in infection, such as core body temperature >38°C or <36°C, heart rate > = 90 bpm, respirations > = 20/min (or partial pressure of carbon dioxide in the blood <32 mmHg), white blood cell > = 12,000/ul or  = <4,000/ul or >10% immature forms. [2] Severe sepsis is marked by acute organ dysfunction (including hypoperfusion and hypotension) that is caused by sepsis [2]. Patient scores on APACHE II and SOFA, which are two widely accepted evaluation systems [42]–[44] based on clinical indictors, were considered important indexes associated with sepsis development and outcome. We focused on patients with pneumonia-induced sepsis and age- and sex-matched healthy controls, and pneumonia was regarded as a common precipitating factor of sepsis [45]. We collected patient information and grouped patients by clinical severity, APACHE II score and SOFA score to examine the genetic factors related to the incidence of sepsis and its clinical presentation. All the patients in our study presented with community acquired pneumonia, not acquired in a hospital or a nursing home residence. Many published papers have examined the relationship between genetic polymorphisms and sepsis. These studies focused on immune cell surface markers [4], [5] and cytokines [6]–[10], which are important for the immune system, and functional genetic polymorphisms. Our work sought to examine functional IFN-γ polymorphisms in relation to pneumonia-induced sepsis.

The function of IFN-γ on infection and inflammation is multifaceted. IFN-γ is produced by most of the immune cells, including NK cells, T lymphocytes and macrophages [46], [47], and is regulated by cytokines IL-12 and IL-18, which are secreted after the antigen is presented [48], [49]. While some experiments have shown that IFN-γ is essential for the host immune response to pathogens [50], [51], the investigation of IFN-γ-deficient patients highlights the functions of IFN-γ in stimulating the inflammatory response and regulating the immune system by demonstrating increased susceptibility to intracellular but not extracellular pathogens, with some decreased immunity as demonstrated by neutrophil mobilization and NK cell activation [52]. However, there are opposing opinions regarding the function of IFN-γ in sepsis following studies in non-human models, including studies in lipopolysaccharide (LPS)-induced shock and cecal ligation and puncture (CLP) models in the rat. These reports stated that IFN-γ could confer susceptibility and affect the outcome. There was evidence of a trend in the plasma level as well as evidence that antibody-mediated blockade of IFN-γ improved the survival rate in an animal model [22]–[24], [53], as IFN-γ deficiency decreases the pathogen burden [53].

The IFN-γ gene has some functional genetic polymorphisms associated with immunologic disease. Following the published literature, we selected four functional point mutations, which included two promoter SNPs (−1616T/C, rs2069705 [31], [34], [54]–[56] and −764G/C, rs2069707 [26]) and two intron SNPs (intron 1, +874A/T, rs2430561 [57]–[59] and intron 3, +3234C/T, rs2069718 [26], [31], [32], [39]). Based on the functional study, the −1616T, −764G, +874A and +3234C alleles were associated with a higher level of IFN-γ than the −1616C, −764C, +874T and +3234T alleles. Our subjects were limited in number, and all were from the Han population of Southern China. No mutation was detected in the −764 position, and the frequencies of heterozygosity at −1616, +874 and +3234 were 0.48, 0.32 and 0.33, respectively, which differed from the frequencies in the National Center for Biotechnology Information (NCBI) data (−1616, 0.499; −764, 0.051; +874, 0.393; and +3234, 0.473). Linkage disequilibrium (LD) analysis show the +874 and +3234 SNPs were in strong LD (r^2^ = 0.894), which partly agrees with surveys from the northern Chinese population [60]. In addition, three haplotypes, CTT, TAC and TTT, were examined.

Although some results were rejected by the corrected Bonferroni analysis, the trend should be noted, such as, −1616T allele conferred protection against sepsis and the +3234C allele was exhibit protection against severity. Compared with those results, some alleles, genotype and haplotype data remained significant after Bonferroni correction, and the significant differences were strong enough to interpret the relationship between sepsis and the IFN-γ polymorphism. The dominate mutation of −1616 and +874 (the addition analysis was the dominant model) were significant associated with the sepsis susceptibility. All the homozygosis mutation of −1616, +874 and +3234 were associated with protection against higher APACHE II and SOFA scores, while the −1616TT genotype was statistic sense in progression to severe sepsis either. As we expected, the haplotype results were consistent with the functional IFN-γ research in sepsis. The CTT haplotype was associated with protection against sepsis initiation but marginal susceptibility to progression to severe sepsis(P = 0.054). The TAC haplotype.was associated with susceptibility to sepsis incidence and protection against progression to severe sepsis. There were three haplotypes for the −1616, +874 and +3234 SNPs. The TAC haplotype composed by three high-expression IFN-γ alleles was the most frequent combination, while the CTT haplotype was the least frequent. Our results suggest that individuals with a genetic background associated with high IFN-γ expression are more susceptible to sepsis, but once they have sepsis they are less likely to experience progression to severe sepsis. Individuals with low IFN-γ expressions are less susceptible to sepsis, which is consistent with the results of the animal experiments.

One limitation of our study is that there were no significant differences in the distribution of IFN-γ SNPs between the sepsis outcomes. The population involved in our study settled in Guangzhou in the subtropical zone, which has a special environment including high temperature, high humidity and air pollution. While the patients in our study had pneumonia-induced sepsis, and some reports suggest that environmental conditions can significantly influence pneumonia [61], [62], we expect our results to be less affected by environment factors because they are difficult to adjust for. As we expected, patients grouped by severity showed significant differences in age and APACHE II and SOFA scores, which corresponds to epidemiologic studies of sepsis, though the mortality from sepsis in our study was 40% compared to the reported rate of 10–20% [1], [3]. Due to this difference, we could conclude that the distribution of IFN-γ SNPs in relation to sepsis outcome is only applicable to our region.

To the best of our knowledge, this is the first study to examine functional IFN-γ SNPs and their haplotypes in relation to the incidence, development and outcome of pneumonia-induced sepsis. In conclusion, our IFN-γ polymorphism study suggests that genetics influence personal sepsis pathology, and IFN-γ SNPs may be used to choose the most appropriate therapy for individuals suffering from sepsis.
